# The League of Mercy

**Published:** 1902-12-20

**Authors:** 


### THE LEAGUE OF MERCY.

The annual meeting of the League of Mercy was held in the board room of St. George's Hospital on the afternoon of the 17th inst. In the unavoidable absence of Sir J. Whittaker Ellis, the chair was taken by Mr. R. C. Antrobus. Among those present were the Marquis Camden, Lord Kilmorey, Lord Mansfield, Lord Farquhar, Lady Grosvenor, Lady Edith King-Noel, Annabella Lady Lamington, Sir Henry Burdett, K.C.B., Lady Burdett, Sir William Collins, Sir Edwin and Lady Hughes, Sir W. and Lady Treloar, Mr. J. Harrison, M.V.O., Lady Faudel Phillips, Rev. Prebendary C. J. Ridgway, Colonel A. A. Owen, Alderman J. Harris, C.C., Colonel Campbell, C.B., Mr. F. C. Antrobus, and others, while apologies for absence were received from many others.

The minutes of the previous annual meeting having been read and approved, the Marquis Camden proposed, and Colonel Campbell seconded, the first resolution:—

"That Mr. E. A. Hambro be appointed chairman, Colonel Arthur Allen Owen deputy-chairman, and Mr. N. Hay-Forbes F.R.C.S.Ed., hon. secretary respectively."

This having been passed unanimously, the second resolution was put to the meeting by Colonel Sir Edwin Hughes, and seconded by Mr. J. Allen Baker, namely:—

"That seven representative members of the Executive Committee be appointed:—The Earl of Mansfield, K.T., the Lord Farquhar, G.C.V.O., Mr. E. A. Hambro, Mrs. R. L. Harrison, Mr. Montague Sharpe, Mr. Seymour Corkran, and Mr. R. C. Antrobus."

This also was carried unanimously. The two last-mentioned members will take the place of the Hon. Mrs. Freeman Thomas and Sir Whittaker Ellis, who have resigned their places on the Executive Committee. In moving the resolution Sir Edwin Hughes spoke of the good that had been done in outlying districts, and the interest in the League that had been awakened by Sir Henry Burdett's illustrated lectures, and he recommended local presidents and vice-presidents to try to have these lectures delivered in their neighbourhood, with a view of drawing the attention of residents to the League and its work. Other speakers also dwelt on the assistance that had been derived from these lectures.

Sir Henry Burdett then gave the financial statement of the year's work. He pointed out that although the year had in many ways been a difficult one for the League, as many givers had elected to send their donations for the sick poor to the Coronation Gift of King Edward's Hospital Fund, the collections yet showed the largest total yet recorded—£8,500—as against £7,000 in 1901, £6,000 in 1900, and £1,000 in 1899. Of the total sum collected it was proposed to give £800 to King Edward's Fund. Sir Henry expressed his hope and confident belief that the money collected by the League would before long reach £20,000. Hitherto the League has been wholly dependent on the services of voluntary workers, and its very nature made it essential that these should be its mainstay; but in view of the rapid growth of the League and the ever-extending radius of its operations, the executive committee had appointed Mr. Reginald Lund permanent secretary. Mr. Lund has great experience in organising, and Sir Henry advised all workers to avail themselves of his services.

There has always been a competition between the metropolitan area and the home counties as to the amount which each contributed to the League. Last year the home counties led by a little, but this year the position has been reversed, thanks chiefly to the splendid work done by Fulham, where the contribution had risen from £450 to £823. In many of the poorer districts also the contributions had increased, and it was evident that the objects of the League were being carried out, and those who could spare only a shilling for the sick poor were willing to give it, if only it were asked from them. They gave the more willingly because they knew that, thanks to the personal voluntary service, of the members of the League, the humblest donor could feel sure that what he gave went to his sick brethren without commission or deduction. The League of Mercy was like the good Samaritan, spontaneously helping the wayfaring man who had fallen by the wayside, and with that spirit animating presidents, vice-presidents, and all the other workers, Sir Henry thought the League might yet aspire to raise as much as £50,000 a year.

Sir Henry dwelt on the good example shown by the City of London, where the aldermen introduced the work of the League to their respective wards, and trusted that the mayors and aldermen of the various suburban borough councils would show themselves as willing to interest their constituents. It was important that a good man or woman should be selected as captain of each street, and the selection of these captains must rest with the local residents.

Sir William Collins, in recounting the year's work of the League, drew attention to the fact that the area of collection of the League of Mercy was larger than the area of distribution of King Edward's Hospital Fund, and that as a stimulus to local interest in the outlying districts the Executive Committee had thought it advisable in some cases to give small donations to local hospitals; but this was never done without personal inspection and visitation, and a careful auditing of the accounts of the institution. Sir William spoke of the successful dance which had lately been held in aid of the League, which had not only provided a sum of £180 wherewith to meet the inevitable expenses of carrying on the work, but had performed the more important function of bringing the League before the notice of many persons who might be willing to work for it.

It has been thought advisable to issue letters of recommendation which the presidents and vice-presidents might distribute to the sick poor within their ken—these letters not to be, like the old "hospital letters," a pledge that the recipient should receive hospital treatment, but only an introduction to the hospital authorities.

Colonel Lockwood dwelt on the difficulty which he found in men collectors, and suggested, that as ladies were generally the best collectors there should no longer be a vice-president and a lady vice-president for each district, but only one vice-president, who might be of either sex. Lord Farquhar supported the motion. Sir Henry Burdett, in opposing the motion, protested against the implication that men were less sympathetic than women, and considered it inadvisable to let them slip out of the work of the League; a change which would, however, require a change in the statute by which the League of Mercy was constituted. Mr. Alderman Harris expressed his agreement with Sir Henry, and stated that in his district (Whitechapel) all the collectors were men. When put to the meeting Colonel Lockwood's motion was lost.

Sir Edwin Hughes expressed the opinion that contributing districts should not be placed on the list in the order of their annual contributions, but in that of their total gifts to the League since they joined it, as local circumstances often affected the subscriptions of a single year.

Votes of thanks to the Chairman and to the authorities of St. George's Hospital for their kindness in granting the use of the Board Room for the meeting terminated the proceedings, after which tea was served, and those present were able to walk through the wards if they wished to do so.

